# mtProtEvol: the resource presenting molecular evolution analysis of proteins involved in the function of Vertebrate mitochondria

**DOI:** 10.1186/s12862-019-1371-x

**Published:** 2019-02-26

**Authors:** Anastasia A. Kuzminkova, Anastasia D. Sokol, Kristina E. Ushakova, Konstantin Yu. Popadin, Konstantin V. Gunbin

**Affiliations:** 1 Center for Mitochondrial Functional Genomics, School of Life Science, Immanuel Kant Baltic Federal University, Kaliningrad, Russia; 2 Center for Integrative Genomics, University of Lausanne, Lausanne, Switzerland; 3 Center of Brain Neurobiology and Neurogenetics, Institute of Cytology and Genetics SB RAS, Novosibirsk, Russia; 4 Novosibirsk State University, Novosibirsk, Russia

**Keywords:** Epistatic interactions, Proteins, Database, Residue solvent accessibilities, Positive selection

## Abstract

**Background:**

Heterotachy is the variation in the evolutionary rate of aligned sites in different parts of the phylogenetic tree. It occurs mainly due to epistatic interactions among the substitutions, which are highly complex and make it difficult to study protein evolution. The vast majority of computational evolutionary approaches for studying these epistatic interactions or their evolutionary consequences in proteins require high computational time. However, recently, it has been shown that the evolution of residue solvent accessibility (RSA) is tightly linked with changes in protein fitness and intra-protein epistatic interactions. This provides a computationally fast alternative, based on comparison of evolutionary rates of amino acid replacements with the rates of RSA evolutionary changes in order to recognize any shifts in epistatic interaction.

**Results:**

Based on RSA information, data randomization and phylogenetic approaches, we constructed a software pipeline, which can be used to analyze the evolutionary consequences of intra-protein epistatic interactions with relatively low computational time. We analyzed the evolution of 512 protein families tightly linked to mitochondrial function in Vertebrates and created “mtProtEvol”, the web resource with data on protein evolution. In strict agreement with lifespan and metabolic rate data, we demonstrated that different functional categories of mitochondria-related proteins subjected to selection on accelerated and decelerated RSA rates in rodents and primates. For example, accelerated RSA evolution in rodents has been shown for Krebs cycle enzymes, respiratory chain and reactive oxygen species metabolism, while in primates these functions are stress-response, translation and mtDNA integrity. Decelerated RSA evolution in rodents has been demonstrated for translational machinery and oxidative stress response components.

**Conclusions:**

mtProtEvol is an interactive resource focused on evolutionary analysis of epistatic interactions in protein families involved in Vertebrata mitochondria function and available at http://bioinfodbs.kantiana.ru/mtProtEvol/. This resource and the devised software pipeline may be useful tool for researchers in area of protein evolution.

## Background

An unevenness of the rates of molecular evolution is typical for the vast majority of functional proteins. A chance of any amino acid fixation in protein depends on their effect on the structure, function and its interactions with other proteins. This results in the observed unevenness in the rates of molecular evolution in different lineages within the same orthologous protein family and in different protein sites in various time intervals. This phenomenon is commonly recognized as heterotachy. Heterotachy is simply the variation in the evolutionary rate of the aligned sites in different parts of the phylogenetic tree, mainly due to epistatic interactions among the substitutions. Epistasis leads to coevolution of various protein regions and even different proteins as well as concerted changes [1–8]. Another interesting feature of protein evolution that relates to heterotachy is called “fixation bursts” - periods of molecular evolution during which very important processes of fixation of the amino acid substitutions unfold over a relatively short period of time [9]. Fixation bursts were observed during analysis of the divergence of mouse and rat [10] and of insertional changes in Drosophila [11]. The above-mentioned fixation bursts can be a result of the process of speciation and/or adaptation to novel ecological niches. Due to the fact that epistatic interactions in proteins are common we can expect that multiprotein complexes can evolve accordingly to fixation bursts: the fixation of one amino acid substitution induces / allows for others etc. Therefore, we decided to uncover such events in evolution of in protein families related to basal cellular functions (e.g. mitochondrial) during the evolution of mammals.

Because of the high complexity of epistatic interactions in proteins, it has been difficult to study the evolution of proteins, especially on deep tree branches. The common pathway to elucidate the evolution of proteins on inner tree branches is ancestral sequences reconstruction (ASR) [12, 13]. The majority of currently available ASR procedures require time reversibility and are based on the general empirical substitution rate models such as WAG [14], LG [15] or JTT [16], which are named after the first letters of their authors’ surnames. However, the main type of protein epistasis - when one mutation interacts with several others, leads to evolutionary irreversibility or to the gradual emergence of restrictive epistatic interactions along the course of protein evolution (or in terms of evolutionary modelling, leads to gradual limitation of general empirical substitution rate model). This in turn makes the highly probable (in terms of general empirical substitution rate model) ancestral state deleterious [1, 3]. Recently various perspective models have been suggested to describe protein evolution in terms of epistatic fitness interactions and/or consequences of epistatic interactions [5, 17], for example, structure-aware CASS model [18]. However, these models are largely inapplicable in large studies due to high computational costs for the protein molecular mechanic and dynamic calculations. The same is true for ancestral protein reconstruction tools that process novel structure and folding stability (e.g. ProtASR [19]), because there is still a lack of experimentally solved 3D protein structures. Another perspective approach which takes into account epistatic interactions in proteins to elucidate their evolution on inner tree branches is the Bayesian mixed (in terms of substitution rates and branch lengths mixing across sequence and along evolutionary time) framework for phylogenetic tree reconstruction, that takes into consideration heterotachy phenomena [20–25]. However, most of those approaches require simultaneous estimation of dozens of parameters, thus the computational effectiveness of such approaches is poor and, due to this obstacle, these approaches are not applicable to large proteome-wide studies.

Nevertheless, the accurate phylogenetic estimation does not necessarily need huge computational efforts. A good example is the usability of CAT protein evolution model, that is entirely based on mixed substitution rate approach. Interestingly, recent comparison between the CAT protein evolution model and the data partitioning with site-homogeneous substitution model clearly demonstrates that the partitioning models are as accurate as the CAT evolution model in spite of >10x computational simplicity [26, 27]. As a very first approximation (without covarion phenomena) the heterotachy phenomena can be modelled via data partitioning, where each partition can have its own evolution rate and its own set of branch lengths. This approach has recently been implemented in a very computationally effective way in IQTree software [28]. Additionally, this year, the branch-unlinked mixture model incorporating heterotachy was implemented also in IQ-TREE software [29, 30]. Thus, it is now possible to analyze the evolution of hundreds of protein families on inner tree branches, while taking into consideration heterotachy, which represents changes in epistatic interactions in proteins - all with reasonable computational time.

Epistatic interactions in proteins are determined mainly by steric and physico-chemical requirements for protein folding in three-dimensional space. Therefore, we assume that the amino acid replacements characterized by large changes in solvent accessibility area, (a measure of solvent exposure of the amino acid in the 3D protein structure) are associated with abrupt changes in protein globule and are expected to be driven by epistatic changes. However, strictly said, such episodes of protein evolution are the consequence (not cause) of epistatic changes and tell nothing new about the mechanism of intra-protein epistasis. In other words, the evolution of residue solvent accessibility (RSA) is anticipated to be tightly linked with changes in protein functions and fitness. Indeed, it was shown that the evolutionary conservation of a protein site correlates with RSA of this site [31–35] and this conservation is additionally linked with the relative site position to the protein active center [31, 32]. At the same time, it was shown that the evolution of natural proteins is often associated with lowering stability against misfolding, which in turn can shuffle parts of the globule with respect to the solvent  [34]. New software and models have been developed to detect positive selection of protein coding genes [33] and proteins [36] based on these observations. Finally, it was recently shown, that the acceleration of mutation fixations in various protein families could fundamentally change the accepted pattern of mutation fixations including permittance of fixations with strong RSA changes [35]. Therefore, for the analysis of the RSA evolution it is necessarily to take into account the family-wise rate of protein evolution. Additionally, it was shown that site-specific evolutionary rates at the level of amino-acids are very similar with such estimations on codon level [36]. Considering this, it is tempting to compare the evolutionary rates of amino acid replacements with the rates of RSA evolutionary changes. This type of comparison may be useful to discriminate nearly neutral changes of protein sequences from changes related to intra-protein epistatic interaction alteration. Moreover, the development of effective phylogenetic computational software tools, such as IQTree software [37], that could accommodate phylogenetic framework into any symbol dictionary allow us to execute ancestral sequence reconstruction procedures so without large computational intensity and inaccuracy (due to usage of standard empirical substitution rate models).

Here, using the robust randomization statistical procedures, heterotachy and site partitioning models within phylogenetic framework, we analyzed the evolution of 512 protein families on inner branches of the tree. The analyzed protein families were selected based on their association with mitochondrial function in vertebrates. We created the first web resource dedicated to analysis of the evolutionary consequences of intra-protein interactions changes in mitochondrial proteome (http://bioinfodbs.kantiana.ru/mtProtEvol/). We showed that site partitioning model, in contrast to heterotachy model, has limited application to the description of RSA evolution. In strict agreement with lifespan and metabolic rate data, we demonstrated that different functional categories of mitochondria-related proteins are subject to selection with accelerated and decelerated RSA evolution rates in rodents and primates. For example, in rodents accelerated RSA evolution is associated with Krebs cycle enzymes, respiratory chain proteins, ROS metabolism and mitochondrial transport, while in Primates these protein functions are stress-response components, mtDNA integrity and translational machinery.

## Construction and content

### Evolutionary analysis pipeline

Our pipeline has three stages: data preparation, jackknifing and data summarizing. Data preparation stage consists of *five* steps. At {*step 1*} we selected 514 HUGO gene names list manually from MitoMiner 4 database [38]. After that {*step 2*} we downloaded (and tested via simple ID concordance test) from ENSEMBL Compara rel. 91 [39] 512 (2 protein names were controversial) protein trees and 512 protein alignments using ENSEMBL REST API [40]. At {*step 3*} we tested all data for heterotachy effects by Procov v. 2.0 software [41] in all tree and in Rodentia-Primata subclades. Using SCRATCH-1D v.1.1 software package [42] at the {*step 4*} we predicted RSA and 8 types of secondary structure for each protein in each protein multiple alignment. We selected this software package for RSA prediction because it is one of the best for structure-aware solvent accessibility prediction [42, 43], and because it allows the user to predict more than three classes of RSA (comparing e.g. with RaptorX_Property_Fast [43]). Detailed RSA prediction allowed us to convert RSA to 20 types and encoded those types in pseudo-amino-acid alphabet (RSA = PAA: -5(unknown) = A, 0 = R, 5 = N, 10 = D, 15 = C, 20 = E, 25 = Q, 30 = G, 35 = H, 40 = I, 45 = L, 50 = K, 55 = M, 60 = F, 65 = P, 70 = S, 75 = T, 80 = W, 85 = Y, 90&95 = V). It should be noted, that we track any reduction in the number of types of amino acid residue, e.g. due to reduction in visible mutation number that is related to the increase in data uncertainty (Supplementary information 1 on the mtProtEvol site). This in turn allowed us to work with PAA in the same way as with canonical amino acids, for example, translating the RSA numeric values to pseudo-amino-acids in protein multiple alignments. At {*step 5*}, using FASTMG software [44], we calculated relative rates of amino acid substitution (REV-model or REV-matrix) and relative rates of pseudo-amino-acid (or RSA) substitutions for each protein family (protein multiple alignment), using likelihood statistics, approximated by PhyML [45].

The construction of matrices containing relative rates of RSA type substitutions for each protein family allowed us to analyze in general the evolution of RSA. In order to perform this analysis we summarized (1) frequencies of RSA type occurrence and (2) ranks of relative rates of RSA type changes from all analyzed protein families (Supplementary information 2 on the mtProtEvol site). We calculated ranks of relative rates of RSA type changes for each RSA type separately, in these calculations we filtered out near zero relative rates of substitution using three thresholds (Supplementary information 2 on the mtProtEvol site). A basic assumption of our approach is that sites in a protein-coding sequence are independent. This assumption is commonly made, and it allows huge simplification of computation, even though it is clear that sites in a protein sequence do not evolve independently. It would be challenging for future studies to include effective computations taking into consideration limited structural constraints [34, 35] related to site dependence.

Jackknifing stage is needed for estimation of branch length variations. For each protein tree analysis we used 100 random delete-half-jackknifed alignments, namely, the pseudo-replicates generated 100 times from the data by random sampling of alignment columns without replacement from the original alignment, each pseudo-replicate being a half of the original alignment. We studied branch length variations using IQTree v 1.6 software because of computational effectiveness [37] and possibility to use both site partitioning model [28] and heterotachy model [29, 30] of protein evolution. The last model is especially important for investigating evolutionary consequences of intra-protein interactions changes (see Background section) in protein evolution, while the first can serve as a baseline for heterotachy model due to model structure. In the both computations, we used (1) the constrained tree topology for each protein family obtained from ENSEMBL Compara rel. 91 and (2) REV-models of amino acids or pseudo-amino-acids relative substitution rates computed at the Data preparation stage. We used the following options for IQTree computations: ‘-m *model* + F*H3’ for analysis using heterotachy model [29, 30] and ‘*model* + F’ for each edge-unlinked (‘-sp’ run option) site partition [28], *model* is the protein family REV-models in PAML text format. We placed alignment sites of both amino acids and pseudo-amino-acids into 8 categories (effectively on average not more than 4 categories (see Utility and Discussion section and Supplementary information 3 on the mtProtEvol site) by simple site diversity measure as described in [46].

Data summarizing stage is intended for robust nonparametric pairwise comparison between branch lengths, based on alignment of amino acid sequences and on alignment of pseudo-amino-acids (or RSA) for the same phylogenetic tree topology of the protein family. The relative (comparing to amino acid replacements rate) increase in the pseudo-amino-acids evolutionary rate can be a hallmark of evolutionary changes, which affect the position of amino acid residues in 3D protein structure (inner residues became outer or vice versa), while the relative decrease of this metric indicates predominant fixations, which preserve the position of amino acid residues in the 3D protein structure. Thus, our metric may suggest new point of view on evolution of proteins, pointing out branches with intensive evolution of 3D protein structure. In order to discriminate these two evolutionary events we went through *six* computation steps. At {*step 1*} we gave each inner tree node a unique label. Then in {*step 2*} we generated two lists of branch lengths for each labelled branch: (*l*_aa_) lengths based on amino acid alignments analysis, and (*l*_rsa_) lengths based on pseudo-amino-acids (or RSA) alignments analysis. In {*step 3*} we calculated in pairs the ratio *L* = *l*_rsa_/*l*_aa_ for each innertree branch of each protein family phylogenetic tree. In {*step 4*} we compared the ln(*L*) value set of each inner tree branch (1-set) to all ln(*L*) values for the all inner tree branches in protein family phylogenetic tree (0-set) using nonparametric U-test (Holm correcting for multiple comparisons [47]) and Cliff’s delta effect size [48]. We used comparable sets in this comparison forming (by random value selection) 0-set size not more than 10 times bigger than 1-set size. We did it in order to select inner tree branches that have significantly higher or lower ln(*L*) values than ln(*L*) values across all branches of the tree or, in other words, to select evolutionary events with relative gain and loss of intra-protein interaction changes. At {*step 5*} we checked the protein names in trees and linking protein names with species names and taxonomy using mygene.info [49] and newick tools [50] with NCBI taxonomy [51]. This allowed us to juxtapose inner tree branches with taxonomic clades and subsequently concentrate our attention on the selected taxonomic clades. At the last {*step 6*} we summarized the results from all proteins under analysis. In doing so we filtered out all near zero internal branches (branch length < 5*10^− 5^) based on RSA and, finally, colorized tree branches with statistically significant accelerated and decelerated RSA change rates.

### Making of the web-interface

In order to give structure to the results, make them interactive and freely available we created a web service (http://bioinfodbs.kantiana.ru/mtProtEvol/), containing all analyzed data and all results. We did this using Apache web server, MySQL 5, Perl 5.24 (CGI module), HTML5, and JavaScript for generating web pages dynamically. We used special applications for interactive multiple sequence alignment (MSAViewer [52]) and for phylogenetic tree visualization (Archaeopteryx.js [53]).

### Construction and analysis of the protein networks

At the last step we summarized our data in protein network framework using STRING [54] and GENEMANIA [55] internet resources. In both resources, for our study we used human datasets as the most functionally annotated dataset. We used Cytoscape 3.5.0 [56] for STRING protein-protein interactions network structure analysis. In this analysis we used three standard measures to characterize the node importance: degree, betweenness centrality (the number of shortest paths that pass through the node), and closeness centrality (reciprocal of the sum of the length of the shortest paths between the node and all other nodes in the graph). For description of protein-specific functional features we used GeneCards resource [57].

## Utility and discussion

With the aim to analyze evolutionary consequences of intra-protein epistatic interactions in mitochondrial proteome we focused on molecular evolution of 512 protein families, involved in mitochondrial function. For this purpose, as well as to take into consideration the importance of RSA evolution in determining intra-protein interaction shifts, we explored the evolutionary rates of amino acid replacements and RSA changes. We compared these rates, using two models of protein evolution: heterotachy and site partitioning, implemented in one software package - IQTree v. 1.6 [37]. Our investigation is split into two parts: first, we analyzed how meaningfulness transitions between all 20 RSA categories are; and, second, we tested the applicability of two models in describing amino acid and RSA evolution; third, we applied best model and investigation strategy for comparative analysis of RSA evolutionary rates in 512 protein families. We did this, using data randomization by jackknife procedure to analyze the sensitivity of results to data variation.

### The reasons for considering 20 RSA categories as a measure of protein evolution

How many categories are enough for evolutionary analysis that is robust to data variation? To answer this question, we analyzed two types of data by random delete-half-jackknifing (see Construction and content section): amino acid residues classified in 20 classes (20 canonical amino acids or 20 classes of RSA), and amino acid residues classified in 8 classes based on protein secondary structure. Obviously, fewer residue classes makes evolutionary analysis more coarse-grained, or, in other words, fewer residue classes makes evolutionary analysis deaf to many mutations. This mutation-deafness in turn should lead (1) to the occurrence of phylogenetic tree branches with zero lengths and (2) to the whole branch lengths shortening. Yes, we demonstrated these two effects when analyzed data based on 8 classes of residues (Supplementary information 1 on the mtProtEvol site). Additionally, using the interquartile range (Q3-Q1) of the ln(*L*) measure for each inner tree branch of each protein family tree, we have shown, that reduction of number of residue type categories to 8 classes, tightly associated with increasing number of tree branches, demonstrates incongruence between length obtained from amino acid alignments analysis, and length obtained from secondary structure alignments analysis (Supplementary information 1 on the mtProtEvol site). Another reason for considering 20 residue classes is the non-zero frequencies of occurrence of all 20 classes of RSA in proteins (Figs. S2.1-S2.2 in Supplementary information 2 on the mtProtEvol site). Thus, the reduction of residue class number is biologically unmeaningful and enhances sensitivity of phylogenetic tree topology to data variation, which in turn leads to an increase in phylogenetic tree uncertainment. Therefore, in this work we track any such reduction of the number of amino acid residue types.

Another interesting question associated with evolutionary analysis of 20 RSA (PAA) categories is “What is the meaning of transitions from one RSA state to another?”. To solve this problem, we carefully analyzed all matrices containing relative rates of RSA type substitutions for each protein family. Summary of these matrices is shown in Supplementary information 2 on the mtProtEvol site. It is of note, that 20 RSA categories include one uncertain RSA category (− 5(unknown) = A), that is useful for protein structure flexibility description and for describing residues, located in disordered protein regions. We demonstrated that this particular RSA category may be substituted by nearly all RSA categories that is in a full agreement with data about disordered protein regions [58]. Frequent substitutions of other RSA categories confined to near nearest RSA states (Figs. S2.3-S2.5 in Supplementary information 1 on the mtProtEvol site), for example frequent substitutions of R category of RSA (corresponds to 0 value of RSA or inner position in protein globule) confined to N, D, C, E, and Q categories of RSA (corresponds to 5–25 values of RSA or inner/intermediate position in protein globule). This is anticipated as usually the evolution of protein 3D structure is quite conservative. However, there are 5 outer RSA categories (S, T, W, Y, and V with values of RSA from 70 to 100) which are characterized by another type of frequent substitutions. In these categories most frequent substitutions to intermediate RSA categories correspond to 15–40 range of RSA values. Once again, it is anticipated, as most outer RSA categories correspond to partially or fully unfolded protein regions, which tend to be folded at least in evolutionary terms. Thus, the classification of RSA in 20 values is biologically meaningful and reflects the nature of protein globules. However, despite of this meaningfulness, it should be mentioned that 20 RSA categories are not natural measures describing protein surface elements and the number of such categories can be changed in order to fine tune the description of peculiar structural properties of specific proteins.

### Comparison amino acid replacements and RSA changes rates based on heterotachy and site partitioning models

**Fig. 1:**
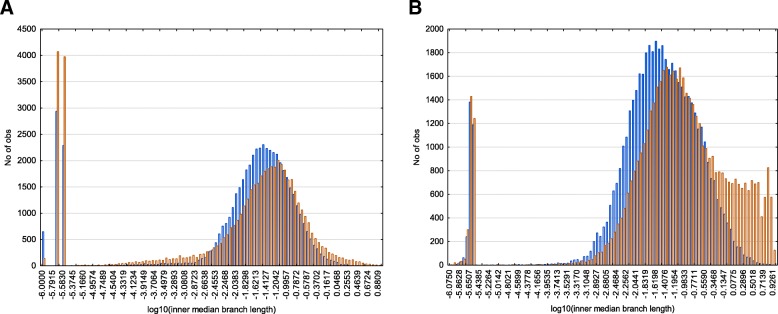
Median length distributions of inner tree branches resulting from heterotachy (**a**) and site partitioning (**b**) models. Colors: blue, trees reconstructed based on amino acid replacements; orange, trees reconstructed based on RSA changes

To check the applicability of models it is necessary to analyze branch lengths distribution. We analyzed the distributions of median lengths obtained by random delete-half-jackknifing procedure on 512 protein families under analysis (see Construction and content section). The variance and asymmetry descriptions of major mode (peak) of the branch length distribution can be used as a proxy for the sensitivity of model [59]. Figure 1 shows that major peak describing branch lengths based on RSA data differ significantly between heterotachy model and site partitioning model: (1) distribution obtained by heterotachy model have smooth thin tails while distribution obtained by site partitioning model have huge right tail containing branches with IQTree hard upper limit of lengths (heterotachy: skewness = − 0.88, kurtosis = 1.81; site partitioning: skewness = − 0.17, kurtosis = − 0.45); (2) distribution obtained by heterotachy model have smaller variance than that obtained by site partitioning model (heterotachy: variance = 0.65; site partitioning: variance = 0.8). Thus, the comparison of data distributions in major peak describing RSA evolutionary changes between heterotachy model and site partitioning model demonstrates that heterotachy model is better suited for RSA evolution description. However, in both models, the branch lengths of RSA-based trees were slightly but significantly (Welch t-test, *p* < 1E-5) higher comparing with amino acid replacement-based trees, indicating that the evolution of RSA is more evolutionary fast process than the amino acid substitutions (we made this comparison only to roughly analyze the relative rates of RSA evolution comparing to amino acid evolution).

**Fig. 2:**
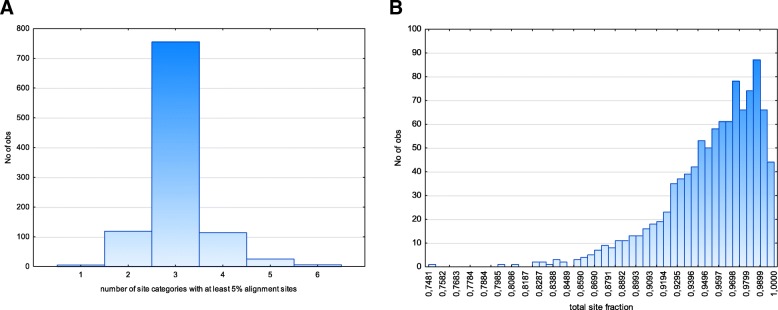
The number of effective (see text) data categories in site partitioning model (**a**) and their cumulative fraction (**b**)

For each class from the defined number (we used 3) of classes of the branch lengths, heterotachy model implemented in IQTree v 1.6 dynamically optimizes residue frequencies, and substitution rates [29, 30]. Site partitioning edge-unlinked model implemented in IQTree v 1.6 have no capacities of site categorization by branch types, however site partitioning model allows manual site categorization [28]. For each defined site category, site partitioning model separately optimizes residue frequencies and substitution rates as in the heterotachy model [28]. Therefore, in order to compare heterotachy model with site partitioning model, it is necessary to implement analogous dynamic categorization of sites. We categorized alignment sites by simple site diversity measure as described in [46]. We fitted the number of site diversity categories (Supplementary information 3 on the mtProtEvol site) so that essential number of these categories (1) should be equal to the number of site classes in the heterotachy model (3 classes) and (2) these essential categories encompass vast majority (> 90%) of alignment. In order to do so we calculated the fraction of alignment sites (in alignments of 512 protein families) belonging to each of site diversity categories (both for amino acid alignments and pseudo-amino-acid alignments), and, after that, calculated the number of categories encompassed et least 5% of alignment sites and total share of alignment sites belonging to these categories. Figure 2 shows that when the number of site diversity categories equals 8, the vast majority of protein families have about 3 (not more than 4) effective site diversity categories in their alignments, that is comparable (by the number of degrees of freedom) to heterotachy model with 3 rates-unlinked site categories and these effective site categories describes more than 90% of alignment sites.

Additionally, we compare site partitioning and heterotachy models in terms of sensitivity of ln(*L*) value (see Construction and content section) to data variation that is simulated by random deletion half-jackknifing.

**Fig. 3:**
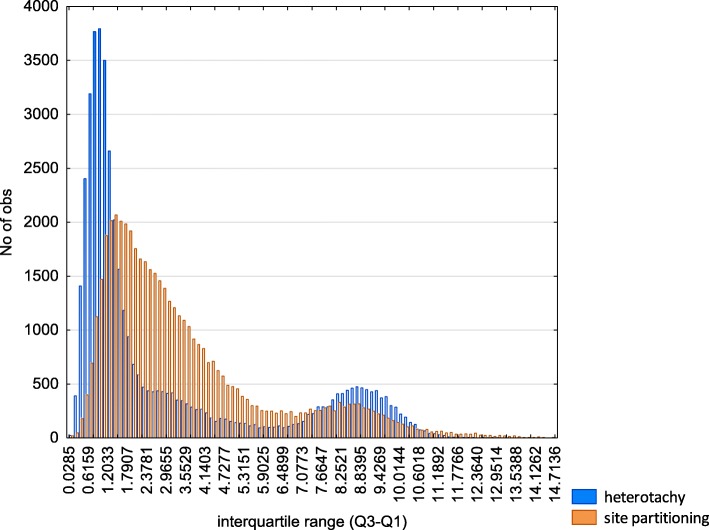
Interquartile range (Q3-Q1) of the ln(*L*) branch measures in heterotachy and site partitioning models

**Fig. 4:**
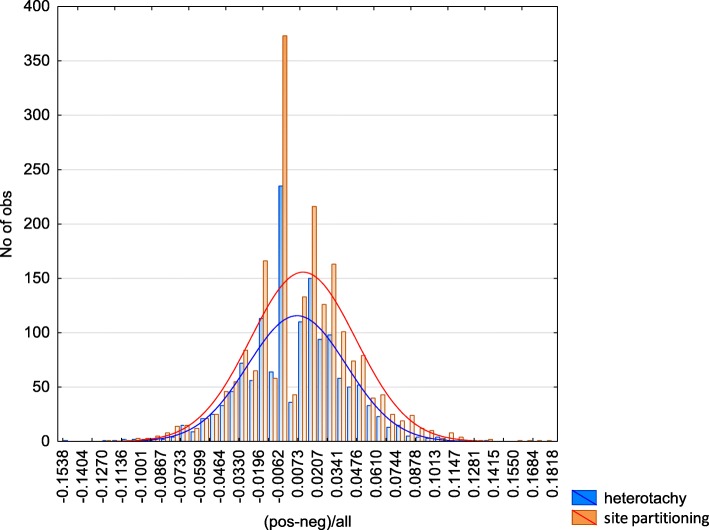
The distribution of differences between the number of branches with positive and negative Cliff’s delta of ln(*L*) (see text) in heterotachy and site partitioning models

**Fig. 5:**
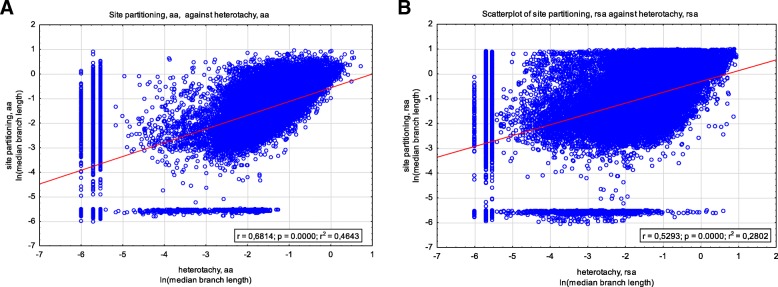
Correlation of natural logarithms of median lengths of inner branches of trees reconstructed using heterotachy and site partitioning models. Correlation of branch lengths in trees reconstructed based on amino acid replacements data (**a**) and on RSA change data (**b**)

1) We calculated the interquartile range (Q3-Q1) of the ln(*L*) measure for each inner tree branch of each protein family tree and analyzed the frequencies of interquartile ranges. Ideally, the jackknife randomization will have no significant effect on the variation the ln(*L*) measure and, evidently, the interquartile range of the ln(*L*) measure for the branch should seek to zero. On the contrary, if interquartile range of the ln(*L*) measure is large enough, then the model is non-robust to data variation and the results should be considered with care and should be adopted by consensus only. The last is strictly a case of site partitioning model (Fig. 3). This is anticipated because the vast majority of protein trees (as well as Primata-Rodentia subtrees) have strong statistical support of heterotachy (covarion), that are observed by Procov 2.0 [41] tests (see mtProtEvol web resource). However, results based on heterotachy model, are characterized by the long right tail of distribution for interquartile ranges of the ln(*L*). Thus, to filter out the majority of possible methodological artifacts we took into consideration only inner tree branches with interquartile range of ln(*L*) lower than 6 (the inflection point, see Fig. 3) for both heterotachy and site partitioning models. We selected this threshold, because the meaningful minimum of branch length is 5E-5 (see Construction and content), therefore the cases, when abs(ln(L)) > 6, reflect the comparisons of smallest branch length with biggest ones. A closer inspection of cases, forming the second peak of interquartile range distribution (both in heterotachy and site partitioning models), have confirmed, that the vast majority of such cases reflect such incongruent or partially incongruent comparison branch lengths (incongruent in terms of the branch length difference in pairwise comparison of results based on amino acids and RSA categories). Additionally, in order to discriminate inequalities in numbers of analyzed branches between heterotachy and site partitioning models after applying various interquartile range thresholds, we calculate the fraction of branches that correspond to the specified threshold for both models. At all checked (4, 5, and 6) interquartile range thresholds the fractions of analysed branches in heterotachy and site partitioning are nearly equal (6: heterotachy, 37,694 branches; site partitioning: 41891; 5: heterotachy, 36,704 branches; site partitioning: 39553; 4: heterotachy, 35,282 branches; site partitioning: 35216). Thus the imposed threshold of interquartile range does not lead to significant inequalities in analyzed branches under heterotachy and site partitioning models.

2) For all 512 protein family trees we calculated the difference between the number of branches with positive and negative Cliff’s delta of ln(*L*) values (see Construction and content section). In other words, in each tree, we calculated the difference between the number of inner branches with accelerated and decelerated RSA evolution. For each protein family tree, we used several Cliff’s delta thresholds (0.3, 0.4, 0.5, 0.6, and 0.7). If there is an asymmetry in distribution of differences between the number of branches with positive and negative Cliff’s delta of ln(*L*) values than the model systematically over- or under-estimate cases with accelerated or decelerated RSA evolution. Both models have symmetrical and normal (KS *p*-value< 0.0001, Shapiro-Wilk p-value< 0.000001) distributions of these differences, thus there are no significant biases in both models in discriminating inner tree branches with faster or slower RSA change rate (Fig. 4). Additionally, this analisis demonstrates that selection of inner tree branches with significantly higher or lower ln(*L*) values comparing to ln(*L*) values of all branches  in the tree is statistically unbiased.

3) Finally, we correlated natural logarithms of median lengths of inner branches between these two models. We constructed two correlations separately for the cases of amino acid replacements study and for RSA change study (Fig. 5). Figure 5 clearly demonstrate that the branch lengths correlation is stronger when the amino acid replacements is under analysis. Moreover, the detailed inspection of branch lengths correlation in the case of RSA evolution (Fig. 5b) demonstrates that phylogenetic trees reconstructed by site partitioning model usually faces on a IQTree hard upper limit of inner branch lengths. Thus, once again we inferred that RSA evolution is poorly modeled by site partitioning model and the results of this model should be considered with care and should be adopted by consensus only.

### Results deposition and user interface

The mtProtEvol, to the best knowledge of the authors, is the first resource that stores the evolutionary consequences of intra-protein interactions changes in mitochondrial proteome. This time only the 512 protein families were annotated in our resource. All protein family-specific information is available through a simple interactive web interface. The results of the analysis are separated in two major groups: individual protein analysis and integrative (by-clade) analysis.

For each analyzed protein family two interactive multiple alignments (multiple alignments of amino acids and RSA values encoded in 20-letter alphabet) and four phylograms (two phylograms for each multiple alignment reconstructed using heterotachy and site partitioning models) were available. The interactivity of alignments and phylograms were provided by MSAViewer [53] and Archaeopteryx.js [54] applications, respectively. The benefits of MSAViewer and Archaeopteryx.js user interfaces are well-known simplicity and usability. Additionally, for each protein family we provided two pairwise tree comparisons, for heterotachy and site partitioning models. We did this comparison in branch-by-branch manner (inner branches only) in order to find branch-outliers with maximum and minimum ln(*L*) values representing cases with accelerated and decelerated RSA changes relative rate (compared to amino acid replacements rate). Other important features deposited for each protein family are heterotachy (covarion) test results obtained by Procov 2.0 [41], results of BLAST screening for sufficient 3D structure homologs in Scratch-1D dataset [42], and various alignment variation features.

The integrative (by-clade) analysis is represented by interactive summary Table (ST) including: taxa (clade) name; frequency of branches with maximum (Na) and minimum (Nd) of ln(*L*) values; lists of proteins (protein families) in which branches are listed in Na and Nb sets; Cliff’s delta thresholds for inner branches selection and Na and Nd sets generation; model of evolution; and protein structure awareness. Clicking on the taxa (clade) name leads to generation of integrative results (GR) across all methods and Cliff’s delta thresholds. These results subdivided into three categories: the number of mitochondrial proteins and species under analysis, the two lists of protein families with accelerated and decelerated RSA evolution. The last two data categories represent: the information about the heterotachy weights (obtained by IQTree v. 1.6 [37]) shown as the ratio between heterotachy weight of RSA changes and heterotachy weight amino acid replacements; *p*-values of heterotachy (covarion); and the measures of model sensitivity (robustness) to data variation for heterotachy and site partitioning models. The model sensitivity measures shown for each protein family. These are the median of ln(*L*) values and shift of mean of ln(*L*) values compared to median of ln(*L*) calculated as (mean-median)/median. The lower the median of ln(*L*) values, the higher the model robustness to data variation is. The higher the shift of mean of ln(*L*) the more the multimodal the distribution of ln(*L*) in protein family is. Working with the GR and ST data accompanied by the ability to generate protein lists for subsequent STRING [54] and GENEMANIA [55] analysis.

### Integrative data analysis on Rodentia and Primates clades

Two clades were selected for detailed analysis. These are Rodentia and Primata, having common evolutionary origin and strictly different ecological strategies (R- and K- respectively), tightly related with housekeeping energetic metabolism carried out by the mitochondrial compartment. Here we describe the integrative results that take into consideration all data as well as data supported by available 3D protein structures only. For in-detail characterization of mitochondrial function evolution we studied two opposite evolutionary cases: cases with accelerated and decelerated RSA evolution (20 RSA categories were used for main analysis; we additionally checked the heterotachy model results using 10 RSA categories (-5(unknown)=A, 0..5=R, 10..15=D, 20..25=E, 30..35=G, 40..45=I, 50..55=K, 60..65=F, 70..75=S, 80..95=W), as a result we found agreement for >90% proteins that were selected based on 20 RSA categories by accelerated RSA evolution – see Supplementary information 4 on the mtProtEvol site). We used the following thresholds and limitations on the results: in heterotachy model cases, Cliff’s delta of ln(*L*) for branch selection is greater than or equal to 0.7 and U-test *p*-value<1E-4 (see Construction and content section); in site partitioning model cases results must agree with the results from heterotachy model, Cliff’s delta of ln(*L*) for branch selection must be greater than or equal to 0.6 and U-test p-value<1E-4.

#### Accelerated evolution of RSA

**Fig. 6:**
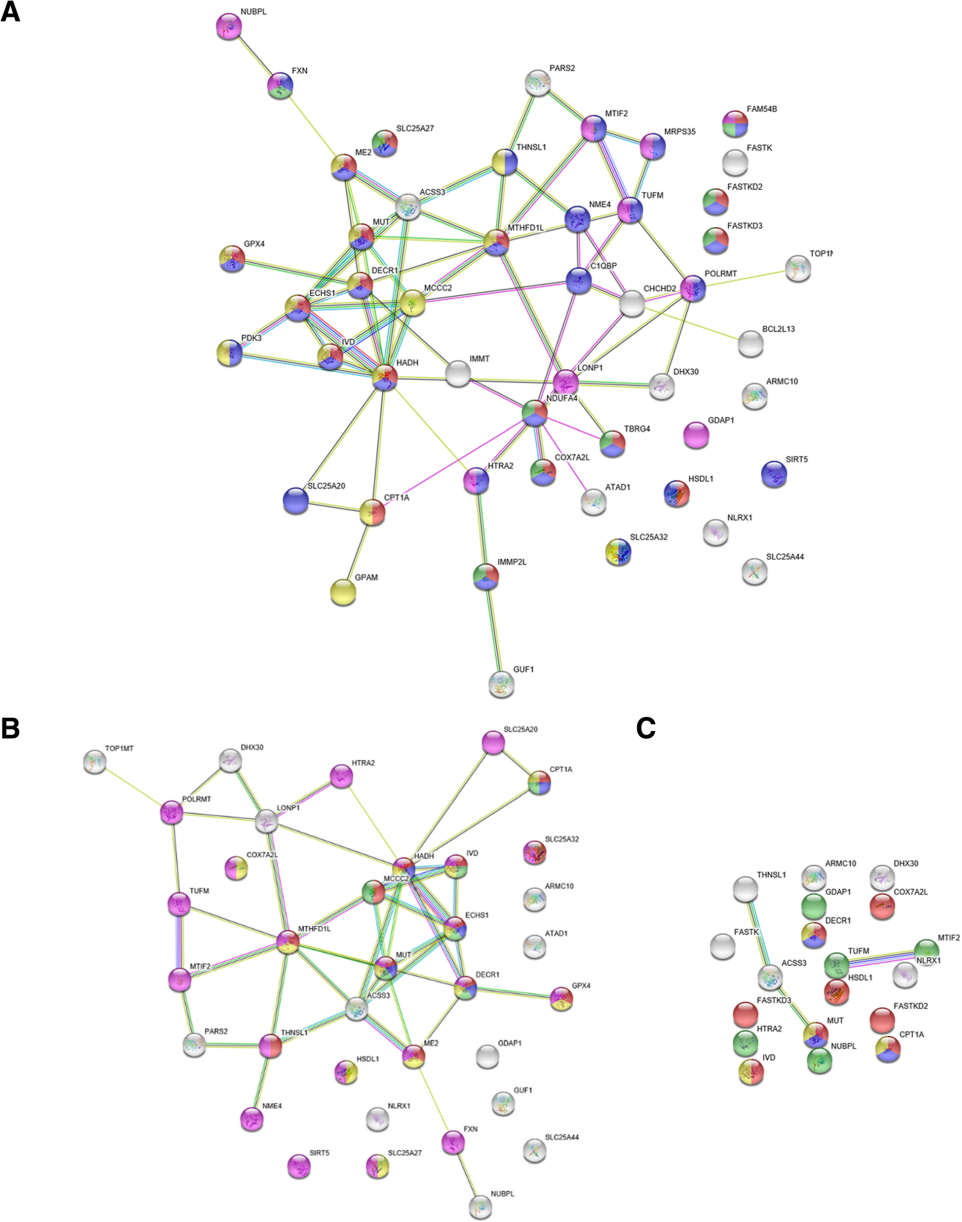
STRING [54] protein-protein interactions for proteins evolved with accelerated RSA change rate on Primata clade branches. **a** analysis using heterotachy model, all data considered; **b** analysis using heterotachy model, protein structure-aware data considered; **c** analysis using heterotachy and site partitioning models, consensus, all data considered

Figure 6 shows STRING protein-protein interaction network topologies composed of proteins evolved with accelerated RSA change rate on Primata clade branches. We obtained these networks using human as a target species. Recall that these cases reflect events of evolutionary changes in intra-protein interactions (see Background section). Note that protein network contains only one cluster (in the cases B and C) surrounded by single proteins. This demonstrates that the majority of genes encoding these proteins co-regulated in human in a coherent manner.

Statistically significant enrichment of protein functions in biological process Gene Ontology (BP GO) category in these networks shown in Tables 1 and 2 in which functional characteristics obtained either based on only proteins evolved with accelerated RSA change rate, in STRING data enrichment, or with 20 nearest (detected by human protein-protein interaction network) proteins, in GENEMANIA data enrichment. Both STRING and GENEMANIA show that the majority of protein-protein interaction network represented by various metabolic functions, especially by fatty acid beta-oxidation components. Additionally to the various metabolic functions the network includes mitochondrial organization components.

**Table 1:** Functional enrichment summary of protein-protein interaction network of proteins evolved with accelerated RSA change rate in Primata clade (based on STRING [54] data; see Fig. 6)

Function	Heterotachy model				Heterotachy and Site partitioning models intersection, all data	
	All data		Protein structure-aware data			
	FDR	Color^a^	FDR	Color^a^	FDR	Color^a^
fatty acid beta-oxidation	0.0001	–	3.9E-05	blue	0.0493	blue
oxidation-reduction process	1.9E-08	red	3.9E-05	yellow	0.0106	red
mitochondrion organization	4.1E-05	violet	0.0002	–	0.0493	green
carboxylic acid catabolism	0.0003	–	3.9E-05	green	0.0493	yellow
carboxylic acid metabolism	3.9E-06	yellow	2.3E-05	red	–	–
single-organism metabolism	2.4E-07	blue	9.0E-05	violet	–	–
small molecule metabolism	0.001	–	0.0002	–	–	–
α-amino acid metabolism	0.009	–	0.0015	–	–	–
cellular respiration	1.0E-06	green	–	–	–	–
negative regulation of RIG-I signaling	0.0102	–	–	–	–	–
mitochondrial transport	0.015	–	–	–	–	–

^a^protein colors shown on the Fig. 6

**Table 2:** Functional enrichment summary of protein-protein interaction network of proteins evolved with accelerated RSA change rate in Primata clade (based on GENEMANIA [55] data; see Fig. 6)

Function	Heterotachy model, FDR		Heterotachy and Site partitioning models intersection, all data, FDR
	All data	Protein structure-aware data	
carboxylic acid catabolism	0.000188	2.53E-12	0.000269
organic acid catabolism	0.000188	2.53E-12	0.000269
small molecule catabolism	0.000812	2.32E-11	0.000768
fatty acid beta-oxidation	0.000812	1.46E-11	0.004036
alpha-amino acid metabolism	0.000888	0.000142	0.008642
monocarboxylic acid catabolism	0.00394	1.46E-11	0.009869
mitochondrial matrix	1.37E-14	3.24E-18	–
mitochondrial nucleoid	9E-06	6.57E-07	–
mitochondrial membrane	2.82E-06	9.28E-06	–
water-soluble vitamin metabolism	0.000812	4.74E-06	–
cellular respiration	5.87E-07	–	0.001263
mitochondrial inner membrane	0.000888	0.00178	–
carnitine transmembrane transport	–	0.003698	0.004036
amino-acid betaine transport	–	0.004428	0.004036
carnitine transport	–	0.004428	0.004036
cofactor metabolism	0.002887	0.006798	–
fatty acid transmembrane transport	–	0.005223	0.004663
quaternary ammonium group transport	–	0.00747	0.006053

In order to analyze protein-protein interaction network for proteins evolved with accelerated RSA changes rate on Primata clade we did standard analysis of network topology by Cytoscape software. For this analysis, we used most full network (Fig. 6a) obtained by analysis of all data using heterotachy model. The results of this analysis are shown in the Table 3.

**Table 3:** Central components of protein-protein interaction network of proteins evolved with accelerated RSA change rates in Primata clade (based on Cytoscape [56] analysis)

Node name	Degree	Betweenness Centrality	Closeness Centrality
HADH	11	0.278204	0.467532
MTHFD1L	8	0.163688	0.428571
ECHS1	7	0.02646	0.395604
NDUFA4	7	0.181265	0.395604
MCCC2	6	0.068717	0.423529
ACSS3	6	0.083957	0.409091
MUT	6	0.049849	0.404494
LONP1	6	0.172386	0.433735
DECR1	6	0.143368	0.423529
TUFM	5	0.077237	0.378947
CHCHD2	5	0.095212	0.367347
C1QBP	5	0.09109	0.4
POLRMT	5	0.099379	0.371134
CPT1A	4	0.096617	0.387097
MTIF2	4	0.032023	0.339623
THNSL1	4	0.06254	0.371134
ME2	4	0.110317	0.336449
IVD	3	0	0.352941
NME4	3	0.024974	0.336449
HTRA2	3	0.107937	0.371134

Let’s compare the molecular functions of some key proteins in the protein-protein interaction network composed of proteins characterized by faster RSA change rate than amino acid replacements rate on Primates clade (Table 3). This network enriched by metabolic functions. The central element of this network is the HADH protein (Hydroxyacyl-CoA Dehydrogenase) that plays an essential role in the mitochondrial beta-oxidation of fatty acids and in pathway of tryptophan utilization. This protein functions in the mitochondrial matrix. ECHS1 protein (Enoyl-CoA Hydratase, Short Chain 1) functions in the two pathways, in mitochondrial fatty acid beta-oxidation and in tryptophan utilization. It has hydratase/isomerase activity and localizes into the mitochondrial matrix as HADH. DECR1 protein (2,4-Dienoyl-CoA Reductase 1) also participates in the fatty acids beta-oxidation. The CPT1A protein (Carnitine Palmitoyltransferase 1A) participates in carnitine-dependent transport across the mitochondrial inner membrane and oxidation of long-chain fatty acids. Co-central element of this network is the MTHFD1L protein (Methylenetetrahydrofolate Dehydrogenase (NADP+ Dependent) 1 Like) that is also located in the mitochondrial matrix and involved in the synthesis of tetrahydrofolate, involved in the purine synthesis. The MCCC2 protein (Methylcrotonoyl-CoA Carboxylase 2) involved in leucine and isovaleric acid catabolism. The ACSS3 protein (Acyl-CoA Synthetase Short Chain Family Member 3) located in membrane (by prediction) and involved in acetate activation. MUT protein (Methylmalonyl-CoA Mutase) involved in the degradation of several amino acids, odd-chain fatty acids and cholesterol. The ME2 enzyme (Malic Enzyme 2) catalyzes the oxidative decarboxylation of malate to pyruvate. The IVD enzyme (Isovaleryl-CoA Dehydrogenase) involves in leucine catabolism. One protein is significantly different from all of the above mentioned. This is NDUFA4 protein (NADH-Ubiquinone Oxidoreductase MLRQ Subunit). It is located in membrane (by prediction) and has NADH dehydrogenase and oxidoreductase activities. It transfers electrons from NADH to the mitochondrial respiratory chain, immediately to ubiquinone.

The most interesting (in evolutionary case) network parts are the LONP1 protein (Lon Peptidase 1, Mitochondrial) a mitochondrial matrix chaperone protein (ATP-dependent serine protease) and POLRMT protein (RNA Polymerase Mitochondrial). The first one mediates the selective degradation of misfolded or damaged proteins. Among its substrates, there is a very important mitochondrial DNA integrity protein helicase TWNK. Additionally it participates in the regulation of mitochondrial gene expression and maintenance of mtDNA because it was shown that it binds to DNA and RNA in a single-stranded, site-specific, and strand-specific manner. The second one (POLRMT) catalyzes the transcription of mtDNA and provides RNA primers for initiation of mtDNA replication. Another interesting network component is TUFM protein (Tu Translation Elongation Factor, Mitochondrial) participating in mitochondrial translation, namely in the GTP-dependent binding of aminoacyl-tRNA to the A-site of ribosomes. The MTIF2 protein (Mitochondrial Translational Initiation Factor 2) also participates in translation initiation promoting formylmethionyl-tRNA binding to the 30S ribosomal subunits.

The protein-protein interaction network of proteins evolved with accelerated RSA rate in Primata have stress-response components. For example, CHCHD2 protein (Coiled-Coil-Helix-Coiled-Coil-Helix Domain Containing 2) translocates from the mitochondrial intermembrane space to the nucleus in response to stress, activates gene transcription under hypoxic conditions, and negatively regulates the mitochondria-mediated apoptosis. The C1QBP protein (Complement C1q Binding Protein) is a multifunctional protein involved in inflammation, ribosome biogenesis, apoptosis regulation, transcriptional regulation, etc. In mitochondria it is involved in translation, namely formation of 55S ribosomes. The NME4 protein (NME/NM23 Nucleoside Diphosphate Kinase 4) participates in the synthesis of nucleoside triphosphates other than ATP, it is also involved in pro-apoptotic signaling by the redistribution of cardiolipin between the mitochondrial inner and outer membrane. Additionally, HTRA2 protein (HtrA Serine Peptidase 2) induces apoptosis by binding the apoptosis inhibitory protein and relocating from endoplasmic reticulum to mitochondria.

Thus, the proteins evolved with accelerated RSA change rate on Primata clade can be characterized as various enzymes participated mainly in fatty acid beta-oxidation, stress-response components and components of mtDNA integrity and protein synthesis machinery.

**Fig. 7:**
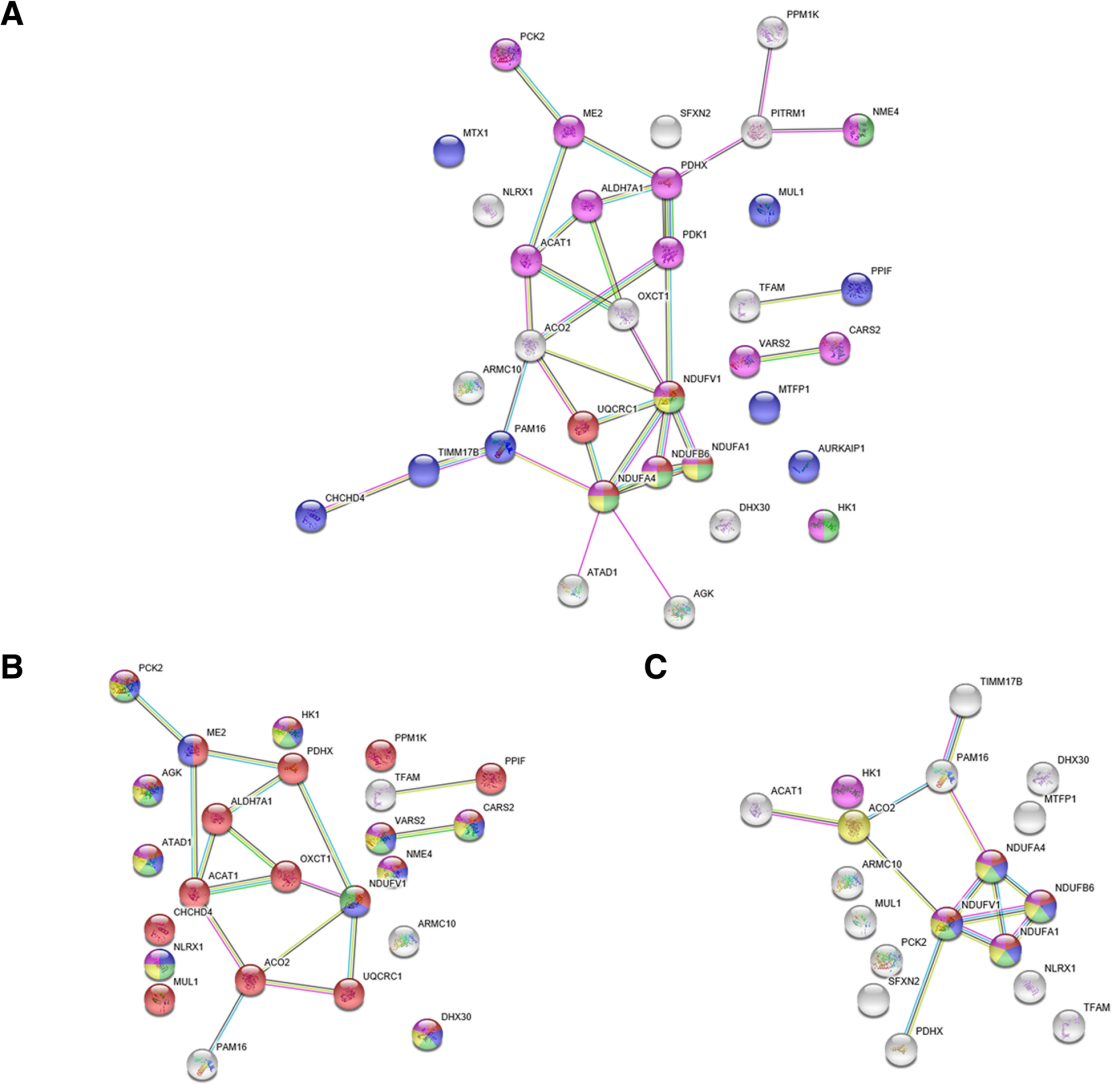
STRING [54] protein-protein interaction for proteins evolved with accelerated RSA changes rate on Rodentia clade branches. **a** analysis using heterotachy model, all data considered; **b** analysis using heterotachy model, protein structure-aware data considered; **c** analysis using heterotachy and site partitioning models, consensus, all data considered

Rodentia clade is also characterized by proteins with accelerated RSA change rate comparing to amino acid replacements rate. Figure 7 shows STRING protein-protein interaction network topologies composed of proteins evolved with accelerated RSA change rates on Rodentia clade branches. We obtained these networks using human as a target species yet again. We select human as a target species because only humans have the most complete and thorough protein interaction dataset in STRING.

Statistically significant enrichment of protein BP GO functions in these networks is shown in Tables 4 and 5 again with functional characteristics obtained either based on only proteins evolved with accelerated RSA changes rate, in STRING data enrichment, or with 20 nearest (detected by human protein-protein interaction network) proteins, in GENEMANIA enrichment. Both STRING and GENEMANIA show that the majority of protein-protein interaction network represented by respiratory chain components, various enzymes, and mitochondrial transport elements. Thus, Rodentia clade is different from Primata clade at least by the accelerated evolution of respiratory chain and mitochondrial transport.

**Table 4:** Functional enrichment summary of protein-protein interaction network of proteins evolved with accelerated RSA change rate in Rodentia clade (based on STRING [54] data)

Function	Heterotachy model				Heterotachy and Site partitioning models intersection, all data	
	All data		Protein structure-aware data			
	FDR	Color^a^	FDR	Color^a^	FDR	Color^a^
mitochondrial ATP synthesis coupled electron transport	0.00036	red	–	–	0.00053	green
mitochondrial electron transport, NADH to ubiquinone	0.00147	yellow	–	–	0.00053	blue
oxidative phosphorylation	0.00331	–	–	–	0.00053	red
ATP metabolism	0.00399	–	–	–	0.00053	violet
cellular respiration	0.00478	–	–	–	0.00053	yellow
oxidation-reduction	0.00615	–	–	–	0.0389	–
small molecule metabolism	0.00147	violet	–	–	0.0493	–
catalytic activity	–	–	5E-06	red	–	–
mitochondrion organization	0.00078	blue	–	–	–	–
purine ribonucleoside triphosphate metabolism	0.00119	green	–	–	–	–
single-organism metabolism	0.00147	–	–	–	–	–
protein targeting to mitochondrion	0.00258	–	–	–	–	–
mitochondrial transport	0.00328	–	–	–	–	–
ketone body catabolism	0.00692	–	–	–	–	–
nucleotide binding	–	–	0.0167	blue	–	–
ribonucleotide binding	–	–	0.0167	green	–	–
purine ribonucleoside binding	–	–	0.033	yellow	–	–
purine ribonucleotide binding	–	–	0.033	violet	–	–

^a^protein colors on the Fig. 6

**Table 5:** Functional enrichment summary of protein-protein interaction network of proteins evolved with accelerated RSA change rate in Rodentia clade (based on GENEMANIA [55] data)

Function	Heterotachy model, FDR		Heterotachy and Site partitioning models intersection, all data, FDR
	All data	Protein structure-aware data	
oxidoreductase complex	3.89E-30	1.17E-21	5.48E-13
mitochondrial membrane	3.06E-26	3.40E-22	9.94E-13
mitochondrial inner membrane	3.06E-26	7.17E-20	1.25E-11
cellular respiration	3.06E-26	7.00E-21	2.81E-10
NADH dehydrogenase (quinone) activity	1.91E-23	2.33E-24	2.91E-09
mitochondrial respiratory chain complex I	4.80E-23	2.87E-24	3.74E-09
oxidoreductase activity, acting on NAD(P)H, quinone or similar as acceptor	1.15E-21	6.05E-23	1.53E-08
mitochondrion organization	5.79E-10	1.62E-09	1.53E-08
energy derivation by oxidation of organic compounds	1.79E-20	6.56E-16	1.31E-07
mitochondrial electron transport, NADH to ubiquinone	2.02E-21	1.44E-22	1.56E-07
oxidoreductase activity, acting on NAD(P)H	9.36E-19	3.95E-20	2.96E-07
mitochondrial ATP synthesis coupled electron transport	1.79E-20	1.17E-21	3.64E-07
respiratory electron transport chain	2.02E-21	5.28E-21	8.52E-07
oxidative phosphorylation	5.21E-25	2.87E-24	1.57E-06
mitochondrial matrix	1.49E-16	3.74E-06	9.09E-15
regulation of acetyl-CoA biosynthetic process from pyruvate	2.19E-10	–	4.13E-06
acetyl-CoA biosynthetic process	6.16E-10	–	7.52E-06
protein targeting to mitochondrion	2.17E-06	–	2.30E-05
pyruvate metabolic process	4.33E-08	–	9.78E-05
thioester biosynthetic process	1.29E-07	–	0.000188
mitochondrial transport	7.79E-06	–	0.000921

In order to analyze protein-protein interaction network in details we again conducted the standard network topology analysis by Cytoscape software. We selected the most complete network (Fig. 7a). The results of this analysis are shown in the Table 6.

**Table 6:** Central components of protein-protein interaction network of proteins evolved with accelerated RSA change rate in Rodentia clade (based on Cytoscape [56] analysis)

Node name	Degree	Betweenness Centrality	Closeness Centrality
NDUFV1	7	0.382456	0.512821
NDUFA4	7	0.273684	0.444444
PDHX	5	0.389912	0.465116
ACO2	5	0.212281	0.454545
ACAT1	4	0.095175	0.392157
OXCT1	3	0.029386	0.384615
NDUFB6	3	0	0.392157
NDUFA1	3	0	0.392157
UQCRC1	3	0.013158	0.408163
PAM16	3	0.202632	0.392157
PITRM1	3	0.194737	0.344828
ME2	3	0.110526	0.377358
ALDH7A1	3	0.022368	0.37037

In protein-protein interaction network of proteins evolved with accelerated RSA change rate on Rodentia clade there are two tightly physically-linked central elements, the NDUFV1 protein (NADH:Ubiquinone Oxidoreductase Core Subunit V1) and NDUFA4 protein (NDUFA4, Mitochondrial Complex Associated), and two peripheral elements, NDUFB6 protein (NADH:Ubiquinone Oxidoreductase Subunit B6) and NDUFA1 protein (NADH:Ubiquinone Oxidoreductase Subunit A1). All these proteins are subunits of core complex I of respiratory chain that transfers electrons from NADH and to ubiquinone. The UQCRC1 protein (Ubiquinol-Cytochrome C Reductase Core Protein 1) is another component of respiratory chain it is a component of the ubiquinol-cytochrome c reductase complex (complex III), it mediates formation of the cytochromes c / c1 complex.

This protein-protein interaction network also has metabolic components. The PDHX protein (Pyruvate Dehydrogenase Complex Component X) is located in the mitochondrial matrix in pyruvate dehydrogenase (PDH) protein complex as a regulatory subunit and acts as linker-enzyme between glycolysis and Krebs cycle by catalyzing the conversion of pyruvate to acetyl coenzyme A. The ACO2 protein (Aconitase 2) catalyzes the second step of the Krebs cycle, the conversion of citrate to isocitrate. The ACAT1 protein (Acetyl-CoA Acetyltransferase 1) is the enzyme plays a crucial role in ketone body metabolism. The OXCT1 protein (3-Oxoacid CoA-Transferase 1) also involves in ketone body metabolism, it catalyzes the transfer of coenzyme A from succinyl-CoA to acetoacetate. The ME2 protein (Malic Enzyme 2) involved in decarboxylation of malate into pyruvate.

One of the most interesting part of this network are the PAM16 protein (Presequence Translocase Associated Motor 16) and PITRM1 protein (Pitrilysin Metallopeptidase 1). First regulates protein translocation into the mitochondrial matrix and plays a role in reactive oxygen species (ROS) homeostasis, while the second is a protease that degrades dissected mitochondrial transit peptides and other unstructured peptides.

Thus, the proteins evolved with accelerated RSA change rate on Rodentia clade can be characterized as a components of respiratory chain, ROS and peptide homeostasis components and enzymes participated in Krebs cycle and ketone body metabolism.

#### Decelerated evolution of RSA

**Fig. 8:**
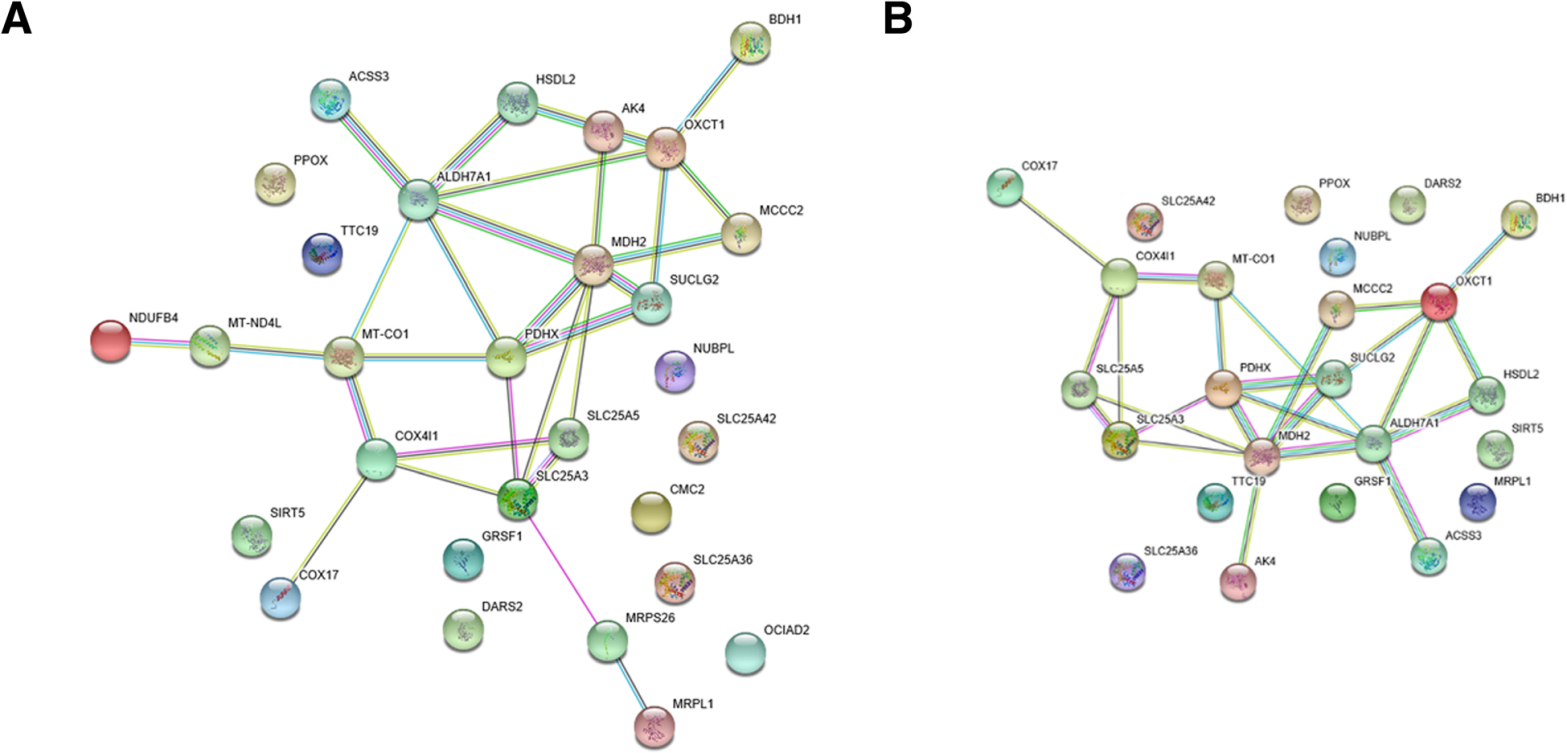
STRING [54] protein-protein interaction for proteins evolved with decelerated RSA changes rate on Primata clade branches. **a** analysis using heterotachy model, all data considered; **b** analysis using heterotachy model protein structure-aware data considered

Figure 8 shows STRING protein-protein interaction network topologies of human proteins evolved with decelerated RSA change rate on Primata clade branches. Recall that these cases reflect events of evolutionary conservation of intra-protein interactions (see Background section). Note that here and below we do not show consensus network obtained by heterotachy and site partitioning model. We do so because these networks have no node connections.

Statistically significant enrichment of protein functions in these networks is shown in Table 7, here only functional characteristics obtained based on GENEMANIA enrichment (with 20 additional nearest proteins) were demonstrated. We did not find any statistically significant (FDR < 0.01) enrichment in BP GO functional categories based on STRING networks constructed both based on protein structure-aware data and on all data. Thus, STRING do not allow us to discriminate biological functions of Primata proteins evolved with decelerated RSA change rate. However, GENEMANIA shows (Table 7) that the majority of protein-protein interaction network represented by respiration and metabolism.

**Table 7:** Functional enrichment summary of protein-protein interaction network of proteins evolved with decelerated RSA changes rate in Primata clade Functional enrichment summary (based on GENEMANIA [55] data)

Function	All data, FDR	Protein structure-aware data, FDR
mitochondrial matrix	7.41E-25	1.85E-20
cellular respiration	7.41E-25	6.65E-20
energy derivation by oxidation of organic compounds	1.18E-20	1.15E-16
mitochondrial inner membrane	3.40E-18	1.28E-15
respiratory electron transport chain	6.77E-16	6.00E-11
hydrogen ion transmembrane transport	1.26E-06	1.20E-10
tricarboxylic acid cycle	1.85E-08	1.47E-06
aerobic respiration	3.07E-07	1.44E-05
oxidoreductase complex	1.70E-10	–
mitochondrial electron transport, NADH to ubiquinone	1.07E-06	–
mitochondrial respiratory chain comp. I	1.26E-06	–
mitochondrial ATP synthesis coupled electron transport	1.73E-06	–
oxidoreductase activity, acting on NAD(P)H, quinone or similar as acceptor	3.86E-06	–
regulation of acetyl-CoA biosynthesis from pyruvate	1.10E-05	–

In order to analyze structure of protein-protein interaction network (Fig. 8a) composed of proteins evolved with decelerated RSA change rate on Primata clade in details we applied Cytoscape software. Some key proteins from this network are shown in the Table 8. There are two key proteins in this network MDH2 (Malate Dehydrogenase 2) and ALDH7A1 (Aldehyde Dehydrogenase 7 Family Member A1). Both proteins are enzymes. First one catalyzes the oxidation of malate to oxaloacetate, second -  metabolizes a number of lipid peroxidation-derived aldehydes and participates in lysine catabolism. The OXCT1 enzyme (3-Oxoacid CoA-Transferase 1), that is involved in ketone body metabolism, evolved with accelerated epistatic interactions changes in rodent clade and decelerated epistatic changes in primates clade. This is also true for the PDHX protein (Pyruvate Dehydrogenase Complex Component X). The SUCLG2 protein (Succinate-CoA Ligase GDP-Forming Beta Subunit) is the enzyme catalyzing reaction of the formation of succinyl-CoA and succinate in the citric acid cycle (TCA).

**Table 8:** Central components of protein-protein interaction network of proteins evolved with accelerated RSA change rate in Primata clade (based on Cytoscape [56] analysis)

Node name	Degree	Betweenness Centrality	Closeness Centrality
MDH2	7	0.295861	0.514286
ALDH7A1	6	0.322876	0.514286
OXCT1	5	0.14488	0.4
PDHX	5	0.140959	0.514286
SLC25A3	5	0.245098	0.461538
MT-CO1	4	0.284314	0.473684
COX4I1	4	0.160131	0.428571
SUCLG2	3	0.035294	0.418605
SLC25A5	3	0.022876	0.428571

There are two components of respiratory chain terminal point in the network. The MT-CO1 protein (Mitochondrially Encoded Cytochrome C Oxidase I) is the terminal component of the respiratory chain that catalyzes the reduction of oxygen to water. The COX4I1 (Cytochrome C Oxidase Subunit 4I1) is another protein from this terminal respiratory chain component.

There are two carriers in the network the SLC25A3 protein (Solute Carrier Family 25 Member 3) catalyzes the transport of phosphate from cytosol into the mitochondrial matrix and the SLC25A5 protein (Solute Carrier Family 25 Member 5) that catalyze the translocation of cytoplasmic ADP from cytoplasm into the mitochondria and ATP from mitochondria into cytoplasm across the mitochondrial inner membrane.

Thus, the list of proteins evolved with decelerated RSA change rate on Primata clade is small and proteins do not characterized by similar functions (except general metabolic function).

**Fig. 9:**
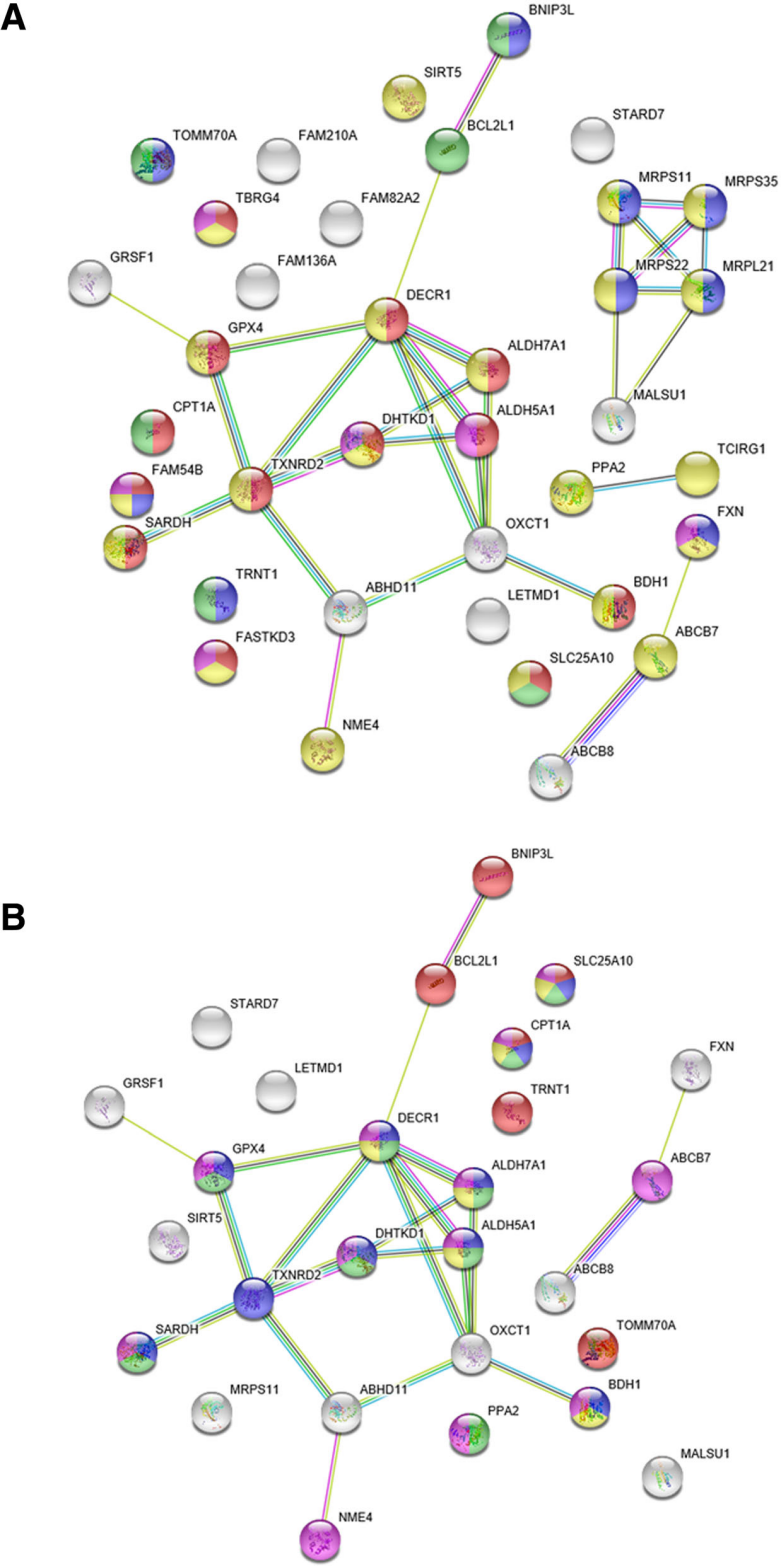
STRING [54] protein-protein interaction for proteins evolved with decelerated RSA change rate on Rodentia clade branches. **a** analysis using heterotachy model, all data considered; **b** analysis using heterotachy model protein structure-aware data considered

Unlike primates, rodents clade characterized by the big number of proteins that characterized by slower RSA change rate comparing to amino acid replacements rate. Figure 9 shows STRING protein-protein interaction network topologies of proteins evolved with decelerated RSA change rate on Rodentia clade branches. We once again obtained these networks using human as a target species. We do so because only humans have the most complete and thorough protein interaction dataset.

Statistically significant enrichment of protein functions in these networks is shown in Tables 9 and 10. The enrichment analyses were done either based on only proteins evolved with accelerated RSA change rate, in STRING enrichment, or with 20 additional nearest (by human protein-protein interaction network) proteins, in GENEMANIA enrichment. Both GENEMANIA and STRING show that the majority of protein-protein interaction network represented by metabolism and mitochondrion organization.

**Table 9:** Functional enrichment summary of protein-protein interaction network of proteins evolved with decelerated RSA change rate in Rodentia clade (based on STRING [54] data)

Function	Heterotachy model			
	All data		Protein structure-aware data	
	FDR	Color^a^	FDR	Color^a^
mitochondrial transport	0.000898	green	0.000486	red
oxidation-reduction process	4.89E-05	red	0.000905	blue
carboxylic acid metabolism	0.00953	–	0.00116	green
small molecule catabolism	0.00953	–	0.00203	yellow
small molecule metabolism	0.0147	–	0.00471	violet
ketone body catabolism	0.0147	–	0.0139	–
negative regulation of mitochondrion organization	0.0276	–	0.021	–
mitochondrion organization	0.000142	blue	–	–
single-organism metabolism	0.000898	yellow	–	–
cellular respiration	0.00151	violet	–	–
organonitrogen compound metabolism	0.0103	–	–	–
mitochondrial translation	0.0147	–	–	–

^a^protein colors on the Fig. 9

**Table 10:** Functional enrichment summary of protein-protein interaction network of proteins evolved with decelerated RSA change rate in Rodentia clade (based on GENEMANIA [55] data)

Function	All data, FDR	Protein structure-aware data, FDR
mitochondrial matrix	3.41E-13	1.44E-11
mitochondrial transport	0.000165	0.002027
mitochondrial membrane	0.000113	0.0022
small molecule catabolism	0.000348	0.0022
protein homotetramerization	0.000191	0.003698
mitochondrion organization	0.000595	0.003698
negative regulation of mitochondrion organization	0.001061	0.048293
mitochondrial outer membrane	0.000348	–

As described above, in order to analyze protein-protein interaction network (Fig. 9a) for proteins evolved with decelerated RSA change rate in Rodentia, we applied Cytoscape software. Central proteins in this protein-protein interaction network are shown in Table 11. There are two key proteins in this network: DECR1 protein (2,4-Dienoyl-CoA Reductase 1), OXCT1 protein (3-Oxoacid CoA-Transferase 1), which are both mitochondrial matrix enzymes. DECR1 is involved in the beta-oxidation and participates in metabolism of unsaturated fatty enoyl-CoA esters; OXCT1 is involved in ketone body metabolism, epistatic changes in its evolution are often observed in both primates and rodents (see above). The DHTKD1 protein (Dehydrogenase E1 And Transketolase Domain Containing 1) is involved in the conversion of 2-oxoglutarate to succinyl-CoA and CO_2_. The ALDH5A1 protein (Aldehyde Dehydrogenase 5 Family Member A1) catalyzes the degradation step of the gamma-aminobutyric acid (GABA) neurotransmitter.

**Table 11:** Central components of protein-protein interaction network of proteins evolved with accelerated RSA change rate in Primata clade, based on Cytoscape [56] analysis

Node name	Degree	Betweenness Centrality	Closeness Centrality
DECR1	6	0.440171	0.619048
OXCT1	5	0.247863	0.541667
TXNRD2	5	0.307692	0.565217
MRPS22	4	0.166667	1
MRPL21	4	0.166667	1
MRPS35	3	0	0.8
MRPS11	3	0	0.8
GPX4	3	0.153846	0.5
DHTKD1	3	0.029915	0.448276
ABHD11	3	0.179487	0.481481
ALDH7A1	3	0.025641	0.481481
ALDH5A1	3	0.025641	0.481481

This network is characterized by the presence of oxidative stress response proteins. The TXNRD2 protein (Thioredoxin Reductase 2) is a pyridine nucleotide-disulfide oxidoreductase which retains thioredoxin in a reduced state, that in turn is a well known key element of oxidative stress response. The GPX4 protein (Glutathione Peroxidase 4) is another oxidative damage protection protein. It protects cells from the toxicity of ingested lipid hydroperoxides. The ALDH7A1 protein (Aldehyde Dehydrogenase 7 Family Member A1) metabolize a number of lipid peroxidation-derived aldehydes, convert betaine aldehyde to betaine and involved in lysine catabolism.

This network is also characterized by the presence of mitochondrial ribosomal protein: MRPS22 (Mitochondrial Ribosomal Protein S22), MRPL21 (Mitochondrial Ribosomal Protein L21), MRPS35 (Mitochondrial Ribosomal Protein S35), MRPS11 (Mitochondrial Ribosomal Protein S11). One of these proteins is a 39S subunit protein (MRPL21), the others are 28S subunit proteins.

Thus, the list of proteins evolved with decelerated RSA change rate on Rodentia clade is characterized by the presence of mitochondrial ribosomal proteins and oxidative stress response components.

## Conclusions and future directions

We constructed a software pipeline, which allowed us to analyze evolutionary consequences of intra-protein interactions changes and implemented all the results into the web resource. W﻿e will regularly update the resource by addin﻿g (at least once a year) new mitochondrial machinery proteins and, also, by adding new quicker methods for finding evolutionary changes in intra-protein epistatic interactions. In our analyses we focused on the RSA change rate normalized by amino acid replacements rate. We have demonstrated, for the first time, that site partitioning model, in contrast to heterotachy model, has limited application for the description of RSA change rate.

We tested our software pipeline on a protein family set, involved into the mitochondrial metabolism. To gain some biological insights we used two model groups of mammals with a common evolutionary ancestor: rodents and primates, different in their level of basal metabolism, body mass, longevity as well as effective population size. We observed that in rodents and primates different categories of proteins were selected towards accelerated / decelerated RSA changes. For example in rodents accelerated RSA evolution has been shown for Krebs cycle enzymes, respiratory chain, ROS metabolism and mitochondrial transport, while in primates these functions were metabolism of fatty acids, stress-response components, translational machinery and mtDNA integrity. Interestingly these categories seem to be in line with ecological strategies of the compared groups: short-lived quickly reproducing rodents optimize protein categories involved in the maintenance of high level of metabolism (respiratory chain, ROS metabolism and mitochondrial transport), while long-lived and slow-reproducing primates optimize the stability of the metabolism (protein synthesis, stress-response components and mtDNA integrity protection). If so, future large scale comparisons of ecologically different mammalian groups may shed light on causes of correlation between the life history traits and functional categories of the most optimized proteins.
